# Genome-wide identification of tomato *B-RRs* gene family and expression analysis in response to ABA and drought

**DOI:** 10.1371/journal.pone.0356487

**Published:** 2026-08-17

**Authors:** Meihua Sun, Jing Li, Yaofeng Zhang, Linlin Tian, Yanxiu Miao, Longqiang Bai, Leiping Hou

**Affiliations:** 1 College of Horticulture, Shanxi Agricultural University, Jinzhong, China; 2 College of Grassland Science, Shanxi Agricultural University, Jinzhong, China; University of Delhi, INDIA

## Abstract

B-type Response Regulators (B-RRs) play a crucial regulatory role in cytokinin signal transduction and are significant for plant development and stress resistance. However, studies on *B-RRs* in tomato are limited. In this study, we conducted a comprehensive analysis of the *B-RRs* gene family in tomato. A total of 10 genes encoding B-RRs transcription factors (named as *SlRR1* to *SlRR10*) were identified. We analyzed the structure, chromosomal localization, phylogeny, protein motifs, and expression levels of these *B-RRs* genes. Gene structure analysis revealed that the *B-RRs* genes in tomato possess between 4–11 exons and 4–12 introns. These genes are distributed across eight chromosomes. Phylogenetic tree analysis classified the *B-RRs* genes into three distinct subfamilies: subfamily I contains 6 genes that share five motifs, subfamily II includes 1 gene that shares three motifs, and subfamily III comprises 3 genes that share four motifs. Expression analysis demonstrated that *SlRR1*, *SlRR2*, and *SlRR5* are highly expressed in the vegetative meristem, while *SlRR3*, *SlRR4*, and *SlRR9* exhibit high expression levels in the roots. *SlRR7* was predominantly expressed in the fruits, whereas *SlRR6*, *SlRR8*, and *SlRR10* show low expression or are undetectable in all tissues. Cis-element analysis revealed the presence of drought and abscisic acid (ABA) response elements in the promoters of the B-RRs genes. Correspondingly, their expression was significantly suppressed by both Polyethylene glycol (PEG) -induced drought stress and exogenous ABA treatment. Given the established antagonism between cytokinin and ABA, our findings suggest that tomato B-RRs may not only play a role in cytokinin signaling but also potentially serve as key nodes in the crosstalk between cytokinin and ABA pathways during drought stress responses. These findings indicate that the *B-RRs* genes in tomato are vital for plant growth, development, and stress resistance.

## Introduction

Cytokinin is a crucial hormone in plants, playing significant roles in promoting cell division as well as regulating the differentiation and growth of plant tissues [1]. Cytokinin B-type Response Regulators (B-RRs) represent a specific class of transcription factors primarily involved in the downstream responses of cytokinin signaling [2]. These factors function by either regulating transcription or interacting with proteins. The N-terminal region of the transcription factor contains the receiver domain (REC) characterized by the conserved D-D-K sequence, while the C-terminal region encom-passes the GARP-like DNA binding domain, also referred to as the MYB-like region [3]. In mouse-ear cress (*Arabidopsis thaliana* L. Heynh) [4], rice (*Oryza sativa* L.) [5], wheat (*Triticum aestivum* L.) [6], and apple (*Malus domestica* Borkh.) [7], 11, 7, 29, and 11 members of the B-RRs family have been identified, respectively. B-RRs play a crucial role in regulating plant growth and development. Studies have demonstrated that the *ARR1* and *ARR12* genes in mouse-ear cress are involved in the regulation of root growth and development [8]. Furthermore, *ARR1*, *ARR10*, and *ARR12* coregulate seed size as well as chlorophyll and anthocyanin synthesis [9]. Additionally, *ARR10*, *ARR12*, and *ARR18* are implicated in the regulation of flower and gametophyte development in mouse-ear cress [10]. In rice, *OsRR21*, *OsRR22*, and *OsRR23* work synergistically to regulate bud and root growth [11]. In apples (*Malus pumila* Mill.), the *MdRRB11* gene promotes flower bud differentiation [7]. *B-RRs* are also involved in the plant’s response to external stress. For instance, *ARR1*, *ARR10*, and *ARR12* in mouse-ear cress enhance drought adaptability [12] and salt tolerance [13] through negative regulatory mechanisms. In wheat, the expression characteristics of *TaARR1* indicate its response to nutrient stress, such as low nitrogen levels [14]. The overexpression of the *ApRR10* gene in perennial ryegrass (*Lolium perenne* L.) has been shown to enhance the plant’s tolerance to salt stress [15]. *B-RRs* also play a significant role in responding to and regulating plant hormone signals. In mouse-ear cress, *ARR1* and *ARR12* can receive cytokinin signals and inhibit auxin transport, thereby affecting cell differentiation in the root elongation region [5]. Furthermore, *ARR1*, *ARR11*, and *ARR12* respond to abscisic acid (ABA) signals and demonstrate increased sensitivity to these signals following mutation [16]. Therefore, *B-RRs* play a crucial role in plant growth, development, stress responses, and hormone signal transduction. Tomato (*Solanum lycopersicum* L.) is an important vegetable crop that is widely cultivated worldwide, holding a significant position in the global agricultural economy. Additionally, it serves as a model plant for fruit research [17]. However, there have been no reports on the *B-RRs* gene family in tomato. The aim of this study was to identify *B-RRs* genes from the tomato genome and analyze their chromosomal locations, promoter cis-acting elements, gene structures, conserved protein motifs, domains, interaction networks, and phylogenetic relation-ships. By examining the expression changes of *B-RRs* in tomato tissues and under various stress treatments, we investigated their potential biological functions, with a particular focus on their role in drought stress and ABA response. This not only establishes a theoretical foundation for elucidating the functions of this gene family but also serves as a reference for other related plant studies.

## Materials and methods

### Whole-genome identification and characterization of B-RRs proteins in tomato

The protein sequences of the mouse-ear cress B-ARRs family were obtained from the mouse-ear cress Information Resource (https://www.arabidopsis.org/) [18] and served as seed sequences. The BLASTP function of the database was employed to search for proteins belonging to the tomato B-RRs family, with an E-value threshold set as 1E-5. Subsequently, protein domains were analyzed using the SMART database [19], where the characteristic domains REC and MYB of the mouse-ear cress B-ARRs family were utilized as criteria to ascertain the membership of proteins within the tomato B-RRs family. Ultimately, a total of 10 members of the tomato B-RRs family were identified.

### Analysis of physicochemical properties, chromosome localization and protein interaction factors of tomato B-RRs family

To analyze the molecular characteristics of the tomato B-RRs family, a variety of bioinformatics tools were employed for systematic analysis. The protein isoelectric point, amino acid composition, and molecular weight were analyzed using the ExPASy platform (http://web.expasy.org/protparam/) [20]. Protein structure prediction was performed using SWISSMODEL (https://swissmodel.expasy.org/) [21] to model the tertiary protein conformation. Subcellular localization information was obtained through the Cell-PLoc system (http://www.csbio.sjtu.edu.cn/bioinf/Cell-PLoc-2/2) [22]. The genome distribution map was generated with the MG2C2.0 tool (http://mg2c.iask.in/mg2c-v2.0/) [23]. The protein sequence of tomato B-RRs was submitted to the STRING database (http://string-db.org) to construct the protein interaction network [24].

### Analysis of phylogenetic tree, gene structure and conserved motif of tomato B-RRs family

The protein sequences of mouse-ear cress, rice, apple, and the tomato B-RR family were aligned using Clustal in MEGA7 software [25], and a phylogenetic tree was constructed based on the Neighbor-Joining method. The tomato B-RRs family has been categorized into three subfamilies based on the classification method used for the B-RRs subfamilies in mouse-ear cress. Protein conserved domain analysis was conducted using the batch CD search function available on the NCBI website (https://www.ncbi.nlm.nih.gov/) [26]. Gene structure analysis was performed by downloading the tomato genome annotation GFF file from the Ensembl website and visualizing the results using TBtools-II [27]. Furthermore, conservative motif analysis was executed with MEME online software (https://meme-suite.org/meme/) [28], where the amino acid sequences of the tomato B-RR family were input to identify and visualize six motifs using TBtools-II.

### Analysis of cis-acting elements in tomato *B-RRs* family gene promoter region

The 2000 bp promoter sequence of the tomato *B-RRs* family gene was downloaded from the Tomato Genome Database (https://solgenomics.net/). Its cis-acting elements were analyzed using the PlantCARE online software (https://bioinformatics.psb.ugent.be/webtools/plantcare/html/) [29], and the results were orga-nized using Excel. Finally, TBtools-II was employed for visual analysis.

### Tissue expression pattern and expression analysis of *B-RRs* family in tomato with different treatments

The analysis of tissue expression patterns was conducted using transcriptome data from the tomato genome database (https://solgenomics.net/). The expression levels (TPM values) of the tomato *B-RRs* family genes in various tissues, including cotyledons, hypocotyls, vegetative meristems, young flower buds, fruits at 10 days post-anthesis, fruits at 20 days post-anthesis, young leaves, mature leaves, and whole roots, were obtained from the database. A heat map was generated using TBtools-II software. The experimental plant material used for PEG-simulated drought and hormone treatments was the ’Zhongshu4’ tomato [30]. The seeds were soaked in warm water and placed in an incubator at 28°C to facilitate germination. The germinated seeds are then seeded in hole trays. Once the seedlings developed two leaves, they were transplanted into nutrient pots and routinely cultivated in the greenhouse. Polyethylene glycol (PEG) is an osmotic agent commonly utilized to simulate drought stress in plants. It achieves this by creating an osmotic potential that decreases water availability to the roots, effectively mimicking conditions of soil water deficit [31]. In the PEG simulated drought treatment experiment, tomato seedlings with four leaves cultivated in soil were selected and subjected to various concentrations of PEG solution (zero, five, ten, fifteen, and twenty percent). The PEG concentration utilized in this article is based on the studies conducted by Muhammad et al. and Wang et al. [32,33]. After a 3-day treatment period, leaves from the same part of the tomato plants were collected and immediately frozen in liquid nitrogen for RNA extraction. Abscisic acid (ABA) is first dissolved in ethanol, followed by the addition of water to prepare a 30 μmol/L ABA solution. Tomato seedlings with four leaves were treated with the ABA solution, while water was used as a control. Tomato leaves were collected at 0, 1.5, 3, 6, 12, and 24 hours post-treatment, and RNA was extracted after rapid freezing in liquid nitrogen. The timing for sample collection following ABA treatment was referenced from the study conducted by Divya et al. [34]. Each treatment included three biological replicates. RNA was extracted from tomato leaves using the DP452 plant RNA extraction kit (Tiangen Biotech, Beijing). The quality of the RNA was assessed through agarose gel electrophoresis, while its purity and concentration were analyzed using the Tecan Infinite M200 enzyme label (Tecan, Mannedorf). cDNA was synthesized using the KR118 reverse transcription kit (Tiangen Biotech, Beijing). The primers utilized for real-time fluorescent quantitative PCR are detailed in Table 1. The real-time fluorescent quanti-tative PCR was conducted using the TB Green fluorescence quantitative kit (Sangon Biotech, Shanghai) within the ABI7500 system, and the results were analyzed with ABI7500 v2.3 software. The ACTIN gene served as the internal reference gene for calculating relative gene expression. Statistical analyses were performed using Microsoft Excel 2016 and SPSS 26.0, while OriginPro 2021 software was employed for mapping.

**Table 1:** Primers for gene expression analysis.

Gene	Gene ID	Forward primer (5′‒3′)	Reverse primer (5′‒3′)
SlRR1	Solyc01g065540	AATTTGCACCACCCTTCCCA	CTGTGCTAGGACCACACCAG
SlRR2	Solyc04g008050	GTGTGGTCTGGTCAGTGGAG	GCTGCCAGTGCTTGAATGTC
SlRR3	Solyc05g014260	AGGGTGTTGTTTCAGTGCCA	TTGTGTGCGATCAACTGGGA
SlRR4	Solyc05g054390	ACAACGTACGAGGGCCAATT	TCGGTAAACCCCAATCCTGC
SlRR5	Solyc06g061030	AAGCCATGGTTGCCTCTACC	ACGGGCAAGACCTTTATCGG
SlRR6	Solyc07g009110	TCAAACATTGGGACAGCGGA	CCGGGTGTATAATCTGCGCT
SlRR7	Solyc08g077230	TGGCCTAATCCGCAACCAAT	CGACGGTGACATGATAGGGG
SlRR8	Solyc11g072330	TCCTGGAAGCCATTCACGAC	GCATTCCAGACGCAATGGTC
SlRR9	Solyc12g010330	GGGCTTGGTGGTTTTCGAAC	GCAGGTGGAACATTTGCGTT
SlRR10	Solyc12g099380	GGGAGATTCGGAAGCAACCA	TCCACAGCTGTTGGGATTCC
ACTIN	Solyc03g078400	GATCAGCGTATCCTTCAGAG	GGCATTGTAGCAGAGAAAAC

## Results

### Identification and physicochemical properties of *B-RRs* gene family members in tomato

**Fig 1:**
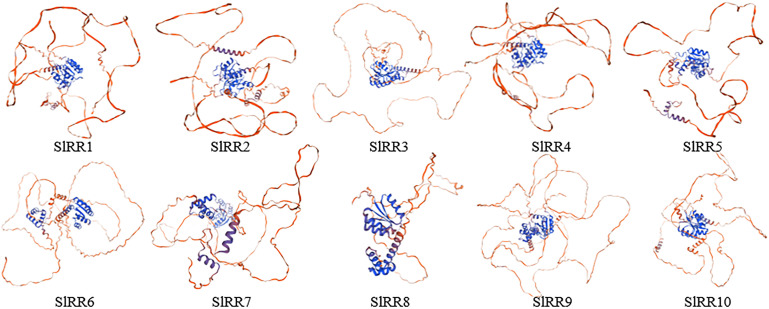
Predicted tertiary structures of B-RRs proteins in tomato. The alpha helix, random coil, and extended strand are represented by blue, yellow and red respectively.

Using the BLASTP function in the tomato genome database, we identified ten *B-RRs* family genes in tomato, designated as *SlRR1* to *SlRR10* based on their chromosomal locations. Physicochemical analysis revealed that the amino acid lengths of the 10 B-RRs proteins ranged from 330 to 708 amino acids, with molecular weights varying from 36.48 to 77.63 kD and theoretical pI between 4.83 and 8.95 (Table 2). Additionally, the instability index of the tomato B-RRs proteins ranged from 37.51 to 57.01, with SlRR3 and SlRR8 exhibiting indices lower than 40, indicating their stability. In contrast, the instability indices of the remaining eight B-RRs proteins were above 40, suggesting relative instability. The negative grand average of hydropathicity values for all B-RRs proteins indicate that they are hydrophilic. Subcellular localization predictions indicated that these proteins are localized in the nucleus. Transmembrane domain analysis revealed that tomato B-RRs proteins lack transmembrane domains (Table 2). Furthermore, tertiary structure predictions indicated that these proteins predominantly consist of alpha helices and random coils, with no transmembrane helices detected (Fig 1, Table 3).

**Table 2:** Information of *B-RRs* gene family members in tomato.

Gene	Gene ID	AA/aa	MW /kD	pI	Instability index	Aliphatic index	GRAVY	SL	TD
SlRR1	Solyc01g065540	677	74.81	6.07	46.22	73.40	−0.596	Nc^a^; *Nc*^b^	0
SlRR2	Solyc04g008050	654	71.62	6.89	57.01	87.13	−0.290	Nc^a^; *Nc*^b^	0
SlRR3	Solyc05g014260	581	65.46	6.16	38.23	78.07	−0.521	Nc^a^; *Nc*^b^	0
SlRR4	Solyc05g054390	663	73.38	5.72	51.05	76.71	−0.529	Nc^a^; *Nc*^b^	0
SlRR5	Solyc06g061030	559	62.30	6.19	51.81	71.34	−0.602	Nc^a^; *Nc*^b^	0
SlRR6	Solyc07g009110	578	65.56	4.83	45.16	76.57	−0.570	Nc^a^; *Nc*^b^	0
SlRR7	Solyc08g077230	560	62.47	5.91	50.40	71.89	−0.639	Nc^a^; *Nc*^b^	0
SlRR8	Solyc11g072330	330	36.48	8.95	37.51	89.76	−0.427	Nc^a^; *Nc*^b^	0
SlRR9	Solyc12g010330	708	77.63	5.82	40.29	70.10	−0.608	Nc^a^; *Nc*^b^	0
SlRR10	Solyc12g099380	555	62.78	5.34	52.78	69.57	−0.600	Nc^a^; *Nc*^b^	0

Note: AA: number of amino acids; MW: molecular weight; pI: theoretical pI; GRAVY: grand average of hydropathicity; SL: Subcellular localization; TD: transmembrane domain; Nc: nucleus; a and b respectively represent the predictions made by CELLO v.2.5 and DeepLoc-2.1 in the subcellular localization section.

**Table 3:** Predicted tertiary structures of B-RRs proteins in tomato.

Gene	Alpha helices/%	Random coils/%	Extended strand/%
SlRR1	16.99	78.14	4.87
SlRR2	16.51	78.9	4.59
SlRR3	16.87	77.97	5.16
SlRR4	15.84	79.64	4.52
SlRR5	23.97	71.2	4.83
SlRR6	17.47	77.34	5.19
SlRR7	22.5	72.68	4.82
SlRR8	25.76	65.15	9.09
SlRR9	15.25	80.15	4.24
SlRR10	16.76	77.12	6.13

Note: The values in each column represent the percentage of the corresponding structural type in the protein.

**Fig 2:**
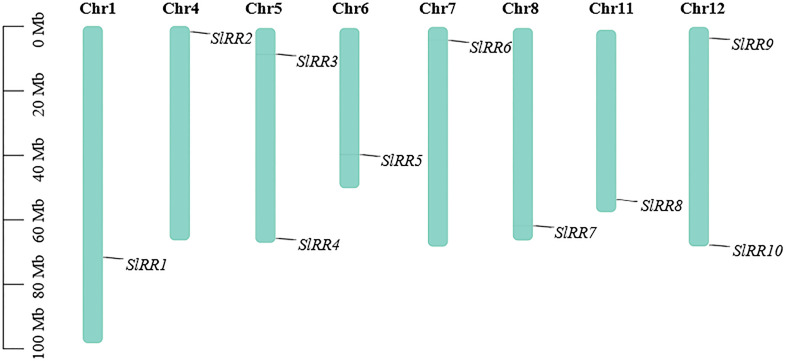
Location analysis of tomato *B-RRs* genes on chromosomes. The figure illustrates the sizes of each chromosome, as indicated by the scale on the left side. Chromosomes 1 to 12 are represented as Chr1 to Chr12.

Fig 2 illustrates the distribution of *B-RRs* genes across the chromosomes of tomato. A total of 10 *B-RRs* genes are located on 8 distinct chromosomes. Chromosomes 1, 4, 6, 7, 8, and 11 each contain one *B-RRs* gene, whereas chromosomes 5 and 12 each contain two *B-RRs* genes. The presence of two *B-RRs* genes on chromosomes 5 and 12 suggests that these chromosomes may have undergone local gene duplication events.

### Protein interaction analysis of tomato B-RRs family

**Fig 3:**
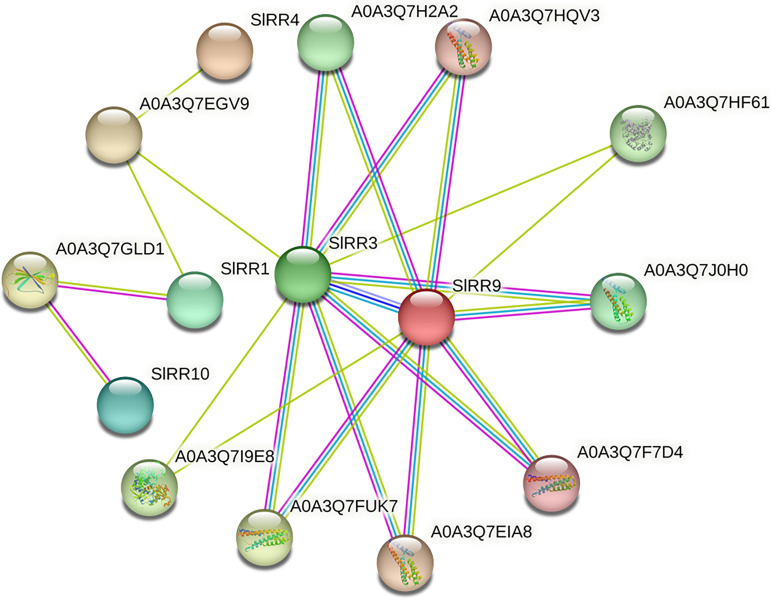
Protein interaction analysis of tomato B-RRs family. The protein-protein interaction network was constructed utilizing the STRING database. Note: A0A3Q7EIA8, A0A3Q7F7D4, A0A3Q7FUK7, A0A3Q7H2A2, A0A3Q7HQV3, A0A3Q7J0H0: HPt domain-containing protein; A0A3Q7EGV9: Acyl transferase 4; A0A3Q7GLD1: WRKY transcription factor 26; A0A3Q7HF61, A0A3Q7I9E8: Indole-3-acetic acid-amido synthetase. The light blue line is from curated database; The pink line indicates experimentally determined; The green line represents textmining; The purple line indicates the protein homology; Blue indicates gene co-occurrence.

The interaction prediction results for the tomato B-RRs family proteins indicated that five B-RRs proteins exhibited interactions with other proteins. The SlRR1 protein interacts with WRKY26 and acyltransferase 4. SlRR3 forms interactions with SlRR9, HPt domain proteins, and indole-3-acetic acid-amino synthase. Additionally, SlRR4 interacts with acyltransferase 4. Beyond its interaction with SlRR3, SlRR9 also engages with HPt domain proteins and indole-3-acetic acid-amino synthase. Furthermore, SlRR10 interacts with WRKY26 (Fig 3).

### Phylogenetic tree, gene structure and conserved motif analysis of tomato B-RR family members

**Fig 4:**
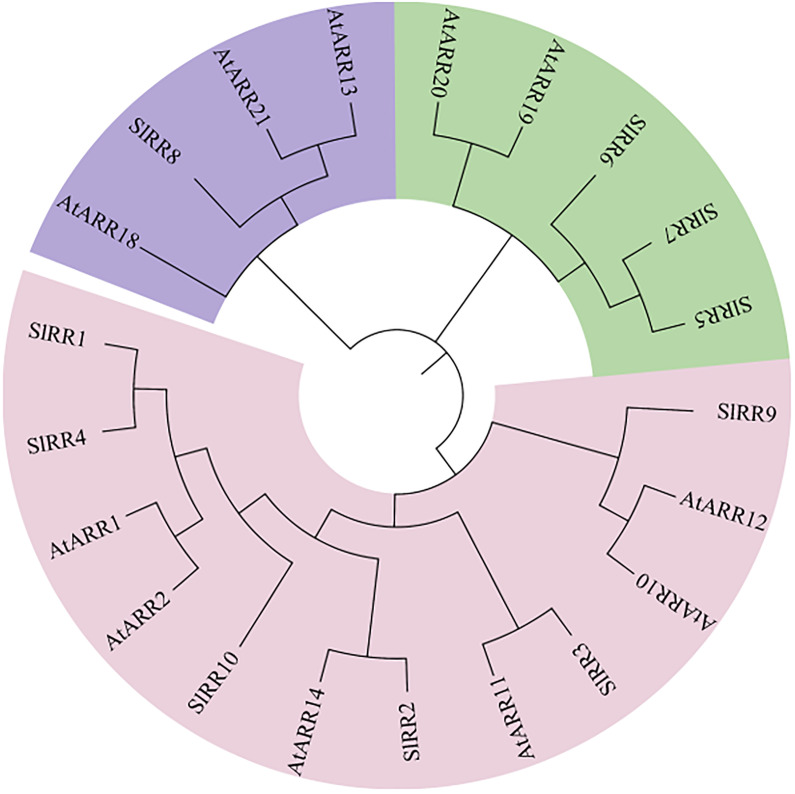
Phylogenetic tree of B-RRs family members of *Arabidopsis* (At), *Oryza sativa* (Os), *Malus domestica* (Md), and *Solanum lycopersicum* (Sl). The subfamilies of the B-RRs proteins are distinguished by different colors: subfamily I is represented in pink, subfamily II in green, and subfamily III in purple.

**Fig 5:**
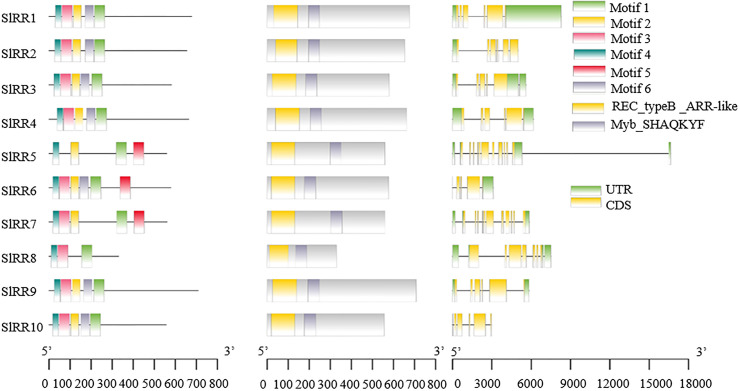
Prediction of B-RRs protein structure in tomato. The gray lines represent introns, while UTR and CDS denote the untranslated region and the coding sequence, respectively. The sizes of the sequences are indicated by a scale at the bottom of the figure.

To investigate the phylogenetic relationship of the tomato B-RRs family proteins, we constructed an evolutionary tree for the B-RRs proteins of mouse-ear cress (11), rice (7), apple(11), and tomato (10). Our results indicate that tomato B-RRs proteins can be classified into three subfamilies: subfamily I, II, and III. Subfamily I includes SlRR1, SlRR2, SlRR3, SlRR4, SlRR9, and SlRR10, while subfamily II comprises only SlRR8. Subfamily III consists of SlRR5, SlRR6, and SlRR7 (Fig 4). Conservative motif analysis revealed that all B-RRs family proteins contained Motif4, suggesting that this motif is a shared characteristic of the family. However, subfamily I is defined by the presence of Motif1, Motif2, Motif3, Motif4, and Motif6. Notably, subfamily II contains Motif1, Motif3, and Motif4. Subfamily III is character-ized by Motif1, Motif2, Motif4, and Motif5 (Fig 5). Furthermore, protein conserved domain analysis confirmed that all tomato B-RRs family proteins possess the B-type ARR-like REC domain and MYB domain (Fig 5). Gene structure analysis, based on tomato genome annotation, demonstrated significant variation in the number of exons and introns among the genes of this family, ranging from 4 to 11 exons and 4–12 introns. Notably, *SlRR5* and *SlRR7* exhibit the most complex gene structures, containing 11 exons and 12 introns, whereas *SlRR6* has the simplest structure, comprising only four exons and four introns (Fig 5).

### Analysis of cis-acting elements in tomato *B-RRs* gene promoter region

**Fig 6:**
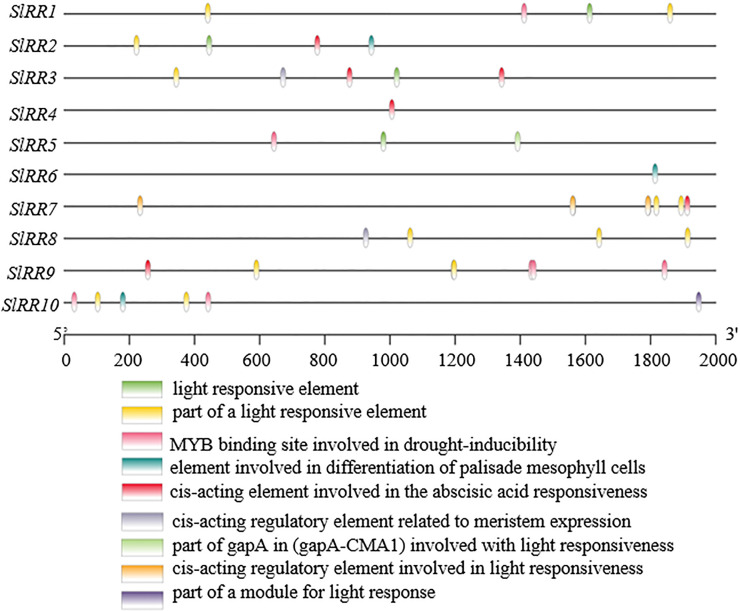
Analysis of cis-acting elements in tomato *B-RRs* promoter region. Predicted putative cis-acting elements were generated using PlantCARE, with different colored bars representing various types of cis-elements. Color legend: green, light-responsive; yellow, part of a light-responsive element; pink, MYB binding site (drought-inducibility); blue, palisade mesophyll cell differentiation; red, ABA-responsive; gray, meristem expression; light green, gapA-CMA1 light-responsive; orange, light-responsive regulatory; purple, module for light response.

The analysis of cis-acting elements within the promoter of the tomato *B-RRs* gene revealed a significant presence of light responsive elements across this gene family. With the exception of the *SlRR4* gene, all members of the *B-RRs* family exhibit light responsive cis-elements. MYB binding site involved in drought-inducibility were identified in the *SlRR1*, *SlRR5*, *SlRR9*, and *SlRR10* genes. Additionally, the *SlRR2*, *SlRR6*, and *SlRR10* genes harbor regulatory elements that are involved in differentiation of the palisade mesophyll cells. Furthermore, the *SlRR2*, *SlRR3*, *SlRR4*, *SlRR7*, and *SlRR9* genes contain cis-acting elements responsive to abscisic acid. Lastly, the *SlRR3*, *SlRR8*, and *SlRR10* genes possess cis-acting elements linked to meristem expression (Fig 6).

### Analysis of tissue expression pattern of tomato *B-RRs* gene

**Fig 7:**
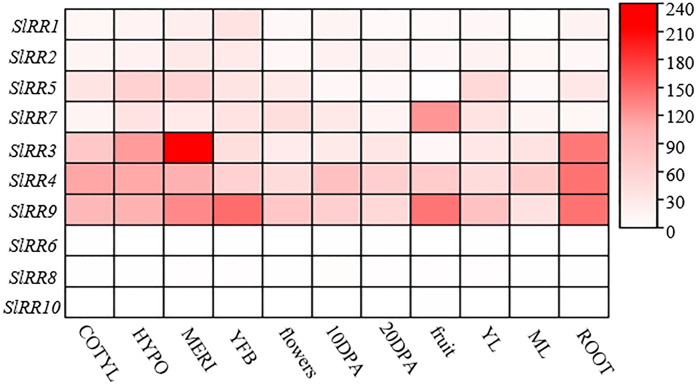
Analysis of expression pattern of *B-RRs* in tomato. COTYL: cotyledons; HYPO: hypocotyl; MERI: vegetative meristems; YFB: young flower buds; 10 DPA:10 days post anthesis fruit; 20DPA:20 days post anthesis fruit; YL: young leaves; ML:mature leaves; ROOT:whole root. The scale of the heat map represents the TPM value.

**Fig 8:**
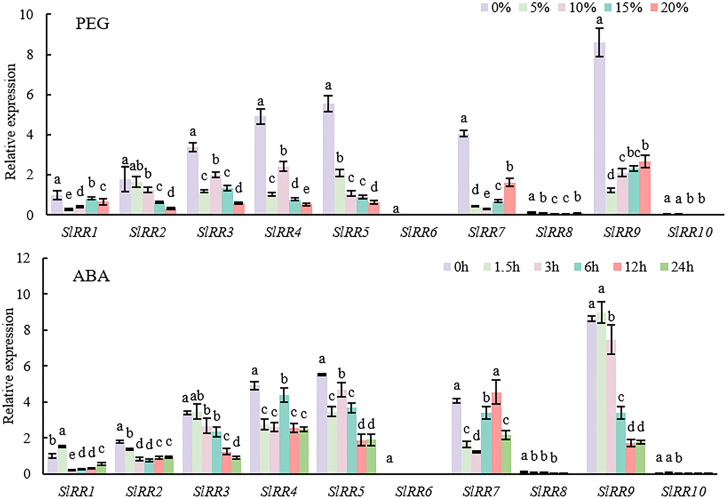
Quantitative RT-PCR verification of *B-RRs* expression levels in different tissues of tomato. COTYL: cotyledons; HYPO: hypocotyl; MERI: vegetative meristems; YFB: young flower buds; 10 DPA: 10 days post anthesis fruit; 20DPA:20 days post anthesis fruit; YL: young leaves; ML:mature leaves; ROOT:whole root.

The expression patterns of *B-RRs* family genes in various tissues of tomato indicate that the *SlRR1* and *SlRR2* genes are highly expressed in the vegetative meristems and young flowers. The *SlRR5* gene exhibits high expression levels in the hypocotyl, vegetative meristems, and young leaves. In contrast, the *SlRR7* gene shows high expression specifically in mature fruit. The *SlRR3* gene is predominantly expressed in the hypocotyl, vegetative meristems, and root tissues. Notably, the *SlRR4* and *SlRR9* genes are expressed abundantly across all tissues, with *SlRR4* showing particularly high expression in roots, while *SlRR9* is highly expressed in the vegetative meristems, young flower buds, mature fruits, and roots. Conversely, the expression levels of the *SlRR6*, *SlRR8*, and *SlRR10* genes are low or undetectable across all examined tissues (Fig 7). Transcriptome data were considered to be reliable after quantitative RT-PCR verification (Fig 8).

### Expression analysis of *B-RRs* gene in tomato under drought stress and hormone treatment

**Fig 9:**
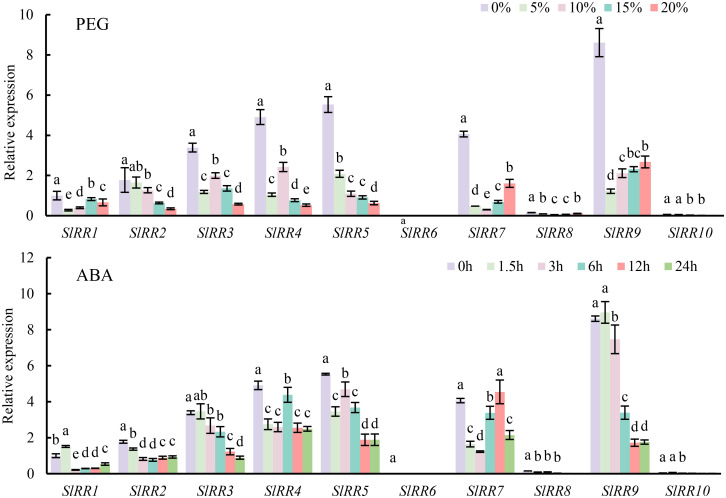
Analysis of *B-RRs* gene expression in tomato treated with PEG and hormone. Lowercase letters indicate significance of differences at the 0.05 level according to Duncan’s multiple range tests.

Given that previous results indicated the presence of drought and ABA response elements in the promoter of *B-RRs*, we conducted an analysis of gene expression changes following PEG-induced drought and ABA treatment to further investigate the functional role of *B-RRs* genes in tomato. The results indicated that the expression of the *B-RRs* gene in tomatoes was significantly down-regulated after PEG-induced drought treatment. Specifically, with increasing PEG concentrations, the expression levels of *SlRR1*, *SlRR7*, *SlRR8*, and *SlRR9* initially decreased and then increased. In contrast, the expression levels of *SlRR2*, *SlRR5*, and *SlRR10* exhibited a continuous decline. The expression levels of *SlRR3* and *SlRR4* decreased initially, then increased, and subsequently decreased again. Notably, *SlRR6* expression was detected exclusively in the zero percent PEG treatment and was absent in all other treatments. Following ABA treat-ment, the expression of the *B-RRs* gene in tomatoes was also significantly down-regulated. The expression of *SlRR1* increased initially before decreasing. The expression levels of *SlRR2*, *SlRR3*, *SlRR8*, *SlRR9*, and *SlRR10* displayed a downward trend. The expression of *SlRR4*, SlRR5, and *SlRR7* decreased initially, followed by an increase, and then a decrease again. Additionally, *SlRR6* gene expression was detected only at 0 hours and was not observed after treatment (Fig 9).

## Discussion

Cytokinin B-type response factors (B-RRs) are crucial regulators of plant growth and development. While the *B-RRs* gene family has been extensively studied in mouse-ear cress, rice, wheat, and apple, research on this gene family in tomato remains unreported [4–7]. The rapid accumulation of biological data and significant advancements in computational performance have greatly enhanced the application of bioinformatics, leading to improved processing efficiency for complex biological problems. Numerous studies have employed bioinformatics methods to identify gene families and predict their functions [35–37] In this study, we systematically identified *B-RRs* family genes in the tomato genome using bioinformatics approaches, analyzing their gene locations, protein structures, evolutionary relationships, and cis-acting elements in the promoter regions. Additionally, we examined tissue expression patterns and the changes in expression levels following drought and hormone treatments.

The number of *B-RRs* genes varies among species, with 11 identified in mouse-ear cress, 7 in rice, 29 in wheat and 11 in apple [4–7]. This study identified 10 *B-RRs* family members, whereas Wang et al. and Liu et al. reported the identification of 12 and 17 *B-RRs* family members in tomatoes, respectively [38,39]. This discrepancy may be attributed to varying standards for E-value thresholds or domain screening during the identification process. The tomato B-RRs proteins are hydrophilic and localized in the nucleus. Among these, SlRR3 and SlRR8 are classified as stable proteins, while the remaining members are considered unstable proteins. Variations in isoelectric point and adipose index were observed, potentially correlating with the functional diversity of these proteins. Protein interaction analysis indicates that the tomato B-RRs protein may interact with proteins containing an HPt domain, acyl transferase 4, and WRKY transcription factor 26, as well as indole-3-acetic acid-amido synthetase. Notably, the WRKY transcription factor is recognized as a crucial regulatory gene for stress resistance. Consequently, it is hypothesized that the B-RRs protein also plays a significant role in the plant’s response to stress [40]. The phylogenetic tree analysis of the B-RRs family in both mouse-ear cress and tomato reveals a similarity between the two, with the tomato B-RRs family being classified into three subfamilies. In *Arabidopsis*, ARR1, ARR2, ARR10, ARR11, ARR12, ARR14, and ARR18 form subfamily I, ARR13 and ARR21 constitute subfamily II, while ARR19 and ARR20 belong to subfamily III [4]. Members SlRR1, SlRR2, SlRR3, SlRR4, SlRR9, and SlRR10 constitute the tomato B-RRs subfamily I, which is characterized by the presence of five conserved motifs. In contrast, members of SlRR8 are classified under the B-RRs subfamily II, which contains three conserved motifs. Additionally, members SlRR5, SlRR6, and SlRR7 are categorized within the B-RRs subfamily III, which is distinguished by four conserved motifs. All tomato B-RRs proteins possess B-type ARR-like REC domains along with MYB domains. The number of introns in the tomato *B-RRs* gene varies from 4 to 12, with the observed variations attributed to the gain or loss of introns. This phenomenon is prevalent in plant polygenic families and holds significant implications for gene functional diversity [41]. Furthermore, the analysis of cis-acting elements in gene promoters enhances our understanding of gene function [42]. In the promoter regions of tomato *B-RRs* family genes, we identified various cis-acting elements, including light responsive elements, MYB binding site involved in drought-inducibility, palisade mesophyll cell differentiation elements, ABA-responsive elements, and meristem expression elements. These findings suggest that this gene family may play a crucial role in plant stress responses as well as growth and development.

The expression of *B-RRs* genes in tomato exhibits significant tissue specificity. Specifically, the *SlRR1*, *SlRR2*, and *SlRR5* genes are highly expressed in the vegetative meristem. It is speculated that the *SlRR1*, *SlRR2*, and *SlRR5* genes may be involved in flower bud differentiation. This functional speculation is supported by conserved roles of their orthologs in other species. For, instance, in mouse-ear cress, *ARR10*, *ARR12*, and *ARR18* collectively determine cell fate in the female reproductive organs [10], while in rice, the *RR21*, *RR22*, and *RR23* genes regulate flower development [9]. Similarly, MdRRB11 promotes flower bud differentiation in apples [7]. This comparative analysis highlights a conserved function of B-RR homologs in reproductive development across various plant species, while also suggesting potential species-specific regulatory nuances, such as the particular tissues or developmental stages in which these homologs exhibit the highest activity. The *SlRR3*, *SlRR4*, and *SlRR9* genes show high expression levels in the root. It is hypothesized that these genes may play a role in the regulation of root development. This hypothesis aligns with the well-established functions of their Arabidopsis homologs; for instance, *ARR1* and *ARR12* are recognized for their roles in regulating root growth and development in mouse-ear cress. A more comprehensive comparison indicates that, although the root-specific expression pattern is conserved, the extent of functional redundancy or specificity among these paralogs may vary between tomato and Arabidopsis, thus warranting further investigation [8]. The SlRR7 gene is predominantly expressed in mature fruits, suggesting that it may play a role in fruit development. This observation suggests a potential species-specific function in the maturation of tomato fruit, a process that is not directly comparable to that of Arabidopsis. However, investigating homologous genes in other fleshy-fruit species, such as rice or apple, could elucidate whether this role represents a Solanaceae-specific innovation or a more general adaptation across different species. In contrast, the expression levels of the *SlRR6*, *SlRR8*, and *SlRR10* genes are low or nearly undetectable across all tissues. This pattern may indicate functional divergence, subfunctionalization, or condition-specific induction—concepts that could be further elucidated by comparing their expression profiles and mutant phenotypes with those of lowly expressed RR homologs in Arabidopsis and rice.

Based on the discovery of drought and ABA response elements on the promoter of the *B-RRs* gene, we analyzed the changes in gene expression following PEG-induced drought and ABA treatment, as well as further investigating the gene functions. Our gene expression analysis revealed that PEG-induced drought treatment significantly inhibited the expression of the *B-RRs* gene in tomatoes, demonstrating that its expression is responsive to drought stress. This conclusion aligns with the presence of drought response elements on the gene promoter, suggesting that the tomato *B-RRs* gene may play a crucial role in regulating drought stress. Previous studies have demonstrated that drought stress suppresses the expression of *B-RRs* genes in mouse-ear cress, and subsequent transgenic experiments confirmed that *ARR1*, *ARR10*, and *ARR12* enhance drought tolerance in mouse-ear cress via a negative regulatory mechanism [12]. Drought stress can induce the accumulation of ABA, and ABA, which antagonizes the function of cytokinin, inhibits cytokinin synthesis, and thereby suppresses the expression of the *B-RRs* gene [16,43]. Additionally, the expression of *B-RRs* genes in tomato was down-regulated following ABA treatment, suggesting that *B-RRs* genes respond to ABA signaling and their expression is inhibited by ABA. This observation is consistent with the presence of ABA response elements in the promoter regions of the *B-RRs* genes in tomato. There exists an antagonistic relationship between abscisic acid and cytokinins in plants [16]. For instance, cytokinins can counteract ABA signals and promote seed germination [44,45] The exogenous application of abscisic acid has been shown to inhibit cytokinin synthesis in plants [46], thereby regulating the expression of cytokinin response factors. This phenomenon has been documented in mouse-ear cress, strawberry (*Fragaria vesca*), Chinese cabbage (*Brassica rapa* ssp. pekinensis), and maize, where ABA inhibits the expression of *cytokinin type A response factors* [45,47–49]. Cytokinin inhibits ABA signaling via the ABI5 transcription factor; conversely, ABA signaling may also inhibit cytokinin signaling through the same transcription factor [50]. In this study, the inhibition of *B-RRs* gene expression by ABA may occur through the ABI5 transcription factor, ultimately diminishing RR-mediated cytokinin signaling. To conclusively establish the role of *B-RRs* in the crosstalk between cytokinins and ABA under drought stress, future studies should directly measure the endogenous levels of cytokinins in tomato plants subjected to drought and ABA treatments. Additionally, employing genetic approaches will be essential to validate the functions of key *B-RRs* genes.

## Conclusion

We identified ten *B-RRs* genes in tomatoes, which are distributed across eight chromosomes and classified into three subfamilies (I, II, and III). The predicted protein localization is in the nucleus. Expression pattern analysis suggests that the *SlRR1*, *SlRR2*, and *SlRR5* genes may play a role in regulating flower bud differentiation, while *SlRR3*, *SlRR4*, and *SlRR9* may be involved in root development. Additionally, *SlRR7* appears to be associated with fruit development. Drought and ABA response elements were identified in the promoter region of the tomato *B-RRs* genes. These genes respond to abiotic stress and hormonal signals, with their expression being modulated by drought stress and ABA.
