# Supplementary material for: Genome-wide identification of tomato B-RRs gene family and expression analysis in response to ABA and drought
Source: PLoS One. 2026 Aug 17;21(8):e0356487. doi: 10.1371/journal.pone.0356487 (PMC13480614; doi:10.1371/journal.pone.0356487)
Supplement: Supplementary file 1 [file pone.0356487.s001.docx]

**Reviewer #1:**

Comment 1‌: In the Materials and Methods section, the authors mention "30 mol/L ABA", which is clearly an unreasonable concentration. Please verify and correct this to a plausible concentration (e.g., µM or nM).

Response‌: Thank you for your careful review and for pointing out the error in the manuscript. The mention of “30 mol/L ABA” in the Materials and Methods section was an unintentional typo. We have corrected this to the appropriate experimental concentration, “30 µM ABA”, in the revised version of the manuscript. We appreciate your valuable comment, which has helped improve the accuracy of our work.

Comment 2‌: The phylogenetic tree in the manuscript was constructed using only Arabidopsis and tomato. The limited species coverage may affect the comprehensiveness and reliability of evolutionary relationship inference. It is recommended to include more representative species to improve the analysis.

Response‌: Thank you for your valuable suggestion. We agree that expanding the phylogenetic analysis to include more species would strengthen the evolutionary inference.

In response to your comment, we have now reconstructed the phylogenetic tree to include B-RR amino acid sequences from additional representative species, specifically rice (Oryza sativa) and apple (Malus domestica). This expanded analysis is included in the revised manuscript and provides a broader perspective on the evolutionary relationships within the B-RR gene family. We believe this revision has significantly improved the comprehensiveness and reliability of our phylogenetic analysis, and we appreciate your insightful feedback.

Comment 3‌: The Discussion section should be strengthened by better linking the findings to existing research. For instance, a more in-depth comparative analysis between the tissue-specific expression of tomato SlRR genes and the functions of their homologs in Arabidopsis and rice would help clarify their conservation and species-specificity.

Response‌: Thank you for the suggestion. We have revised the Discussion to include a detailed comparative analysis of tissue-specific expression among tomato, Arabidopsis, and rice homologs, clarifying their conservation and potential species-specific roles.

Comment 4‌: The language is not fluent and contains numerous grammatical and wording errors. For example:

o Line 98: "Polesitilegicle (PEG)" should be "Polyethylene glycol (PEG)".

o "Tran-scription factors" should be "Transcription factors".

o It is recommended that the entire manuscript undergoes professional English editing or proofreading by a native speaker.

Response‌: Thank you for identifying these language issues. We have corrected the specific errors noted and have now had the entire manuscript professionally edited to improve its fluency and grammar.

Comment 5‌: Figure 1 requires the addition of confidence scores (e.g., from the modeling software) to clarify the reliability of the predicted structures.

Response‌: Thank you for your suggestion. We have revised Figure 1 to include the requested confidence scores, with the detailed score distribution provided separately in the newly added Table 3.

Comment 6‌: Figure 7 needs specific scale annotation for the TPM value range. The color gradient should be optimized to improve readability.

Response‌: Thank you for your suggestion. We have now optimized Figure 7 by adding a specific scale annotation for the TPM value range and improving the color gradient to enhance overall readability.

Comment 7‌: The explanation "lowercase letters indicate significance" in Figure 8 is insufficient. The specific statistical method used should be explicitly stated.

Response‌: Thank you for your suggestion. We have updated Figure 8 to clearly state the specific statistical method used for the significance analysis.

Comment 8‌: The image resolution is too low, resulting in blurred text.

Response‌: Thank you for pointing out the issue with image clarity. We have regenerated and replaced all figures in the manuscript with high-resolution versions. The slight blur in the PDF may have been caused by file compression during formatting; we suggest downloading the original image files for a clearer view, as they are of high resolution.

Comment 9‌: In Table 2, what does "Nc a ; Nc b" represent? Please define these abbreviations in the table note to avoid ambiguity.

Response‌: Thank you for your feedback. We have now added a clear definition for "Nc a" and "Nc b" in the notes of Table 2 to resolve any potential ambiguity.

Comment 10‌: Lines 10-12, the statement "11, 7, 29, and 11 members" is vague. It is recommended to explicitly link the numbers to their respective species (Arabidopsis, rice, wheat, apple) and corresponding reference numbers, for example: "11, 7, 29, and 11 members have been identified in Arabidopsis [4], rice [5], wheat [6], and apple [7], respectively."

Response‌: Thank you for this suggestion. We have revised the sentence to clearly link each member count to its corresponding species and reference, as recommended.

Comment 11‌: In the Keywords, "ABA response" and "Drought stress" are suggested to be adjusted to "ABA responsiveness" and "Drought tolerance" respectively, to align with common terminology in the field.

Response‌: Thank you for the suggestions. We have updated the keywords as recommended, changing "ABA response" to "ABA responsiveness" and "Drought stress" to "Drought tolerance" to align with the field's standard terminology.

Comment 12‌: The formatting of reference citations is inconsistent; for instance, some references lack page numbers or volume/issue numbers.

Response‌: Thank you for your feedback. We have revised the reference list in accordance with the journal's formatting guidelines to ensure consistency, including the addition of missing volume, issue, and page numbers where applicable.

**Reviewer #2:**

Comment 1‌: The Abstract section might be confusing and should be reorganized. For example, its first sentence states a role for B-RRs gene family in cytokinin signal transduction, while its findings further demonstrate its involvement in ABA-mediated drought stress responses. Given the known antagonistic relationship between ABA and cytokinin, we are curious whether the authors have considered a potential role for this family in the crosstalk between these two hormonal pathways? It is essential to determine the cytokinin content in tomato plants under drought stress and ABA treatment conditions.

Response‌: Thank you for your insightful and constructive comment. In response, we have thoroughly revised the manuscript:The Abstract has been reorganized to present the rationale and findings more logically. A new section has been added to the Discussion. Here, we explicitly explore the potential role of the studied B-RRs in mediating the crosstalk between cytokinin and ABA signaling during drought stress, based on our expression pattern analysis and the established antagonistic relationship between the two hormones.Regarding your suggestion on cytokinin content measurement, we fully agree that it would be a valuable experiment. We plan to perform these measurements in our follow-up functional studies. In the revised discussion, we have incorporated this point as an important future research direction.

Comment 2‌: The following aspects of the bioinformatically predicted results necessitate further validation through molecular experiments. Firstly, the subcellular localization predictions should be experimentally validated, for example, by transiently expressing GFP-fusion constructs in protoplasts or tobacco leaves. Secondly, the tissue-specific expression pattern identified by RNA-seq needs to be verified by RT-qPCR (Fig.7). Thirdly, the coding sequences (CDS) of the ten SlRR genes should be cloned and validated through Sanger sequencing. Additionally, the cloned SlRR CDSs should be deposited in public database, such as GenBase/NGDC (https://ngdc.cncb.ac.cn/genbase) and GenBank/NCBI (https://www.ncbi.nlm.nih.gov/genbank/).

Response‌: Thank you for these constructive suggestions to strengthen the validation of our results.

In direct response to your recommendation, we have now performed RT-qPCR analysis, which successfully validates the tissue-specific expression patterns originally observed in the RNA-seq data (Fig. 7). These new results have been added to the revised manuscript.

We fully agree that experimental validation of subcellular localization and cloning of the CDS sequences, as you suggested, are important next steps. Given the current constraints of project timeline and resource availability—particularly nearing the conclusion of this study—we were unable to incorporate these additional experiments at this stage. However, we have explicitly acknowledged these points as key objectives for our future research in the revised Discussion section.We appreciate your guidance, which has significantly improved the present work and clearly outlines the path forward.

Comment 3‌: In the sentence ‘The protein sequences of the Arabidopsis B-ARRs family were obtained from the Tomato Genome Database (https://solgenomics.net/)’ (Line46-47), the source was incorrectly cited as ‘the Tomato Genome Database (https://solgenomics.net/)’ instead of the correct database, ‘the Arabidopsis Information Resource (TAIR, <https://www.arabidopsis.org/)’.>

Response‌: Thank you for identifying this oversight. The sentence has been corrected to accurately cite the source: "The protein sequences of the Arabidopsis B-ARRs family were obtained from The Arabidopsis Information Resource (TAIR, [https://www.arabidopsis.org/)."](https://www.arabidopsis.org/).\")

Comment 4‌: ‘an E-value threshold set as 1E-5’ was improperly written as ‘an E-value threshold set as -5’ (Line49).

Response‌: Thank you for pointing out this error. The text has been corrected to "an E-value threshold set as 1E-5" in the revised manuscript.

Comment 5‌: Is the multiple sequence alignment (MSA) performed using ClustalW or MUSCLE, as implemented in the MEGA7 packages? (The relevant text states, ‘The protein sequences of the Arabidopsis and the tomato B-RR family were aligned using MEGA7 software’, Line69-70).

Response‌: Thank you for raising this point. We have clarified the Materials and Methods section to specify that the multiple sequence alignment was performed using the ClustalW method within the MEGA7 software.

Comment 6‌: Please provide the website address for the NCBI website (https://www.ncbi.nlm.nih.gov/Structure/cdd/wrpsb.cgi, Line74), MEME online software (https://meme-suite.org/meme/, Line77), and PlantCARE online software (https://bioinformatics.psb.ugent.be/webtools/plantcare/html/, Line84).

Response‌: Thank you for your suggestion. We have updated the manuscript to include the full website addresses for the NCBI CD-search, MEME, and PlantCARE online tools.

Comment 7‌: It is suggested that the B-RR genes from Oryza sativa be incorporated into the NJ-tree (Fig.4). Bootstrap sampling should be employed to construct the NJ-tree, and the bootstrapping values should be indicated on its branches.

Response‌: Thank you for your valuable suggestion. We have now revised the phylogenetic tree (Fig. 4) by incorporating B-RR genes from Oryza sativa, and the bootstrap support values are indicated on the branches.

Comment 8‌: The common name, such as Thale cress or mouse-ear cress, should be used as an alternative to the non-italicized “Arabidopsis” (e.g., Line10, Line14, Line18, Line27, Line46, Line219, Line289, Line301, etc). This is necessary because Arabidopsis is a genus name and should be written in italics. Additionally, the species name Arabidopsis thaliana (Line246-247) should also be italicized to adhere to standard taxonomic converntions.

Response‌: Thank you for pointing this out. We have revised the manuscript throughout to ensure correct formatting.
