# Supplementary material for: Genome-wide identification of tomato B-RRs gene family and expression analysis in response to ABA and drought
Source: PLoS One. 2026 Aug 17;21(8):e0356487. doi: 10.1371/journal.pone.0356487 (PMC13480614; doi:10.1371/journal.pone.0356487)
Supplement: Supplementary file 2 [file pone.0356487.s002.doc]

Response to Reviewers

Manuscript Title: Genome-Wide Identification of tomato B-RRs Gene Family and Expression Analysis in Response to ABA and Drought

Manuscript ID: PONE-D-25-29384R1

**Reviewer #1:**

**Comment 1:** The manuscript currently uses both "Fig 1" and "Fig. 1". It is recommended to choose one format and apply it consistently throughout (e.g., "Fig 1" or "Figure 1").

**Response:** Thank you for pointing out this inconsistency. We have now carefully reviewed the entire manuscript and unified all figure citations to "Fig." (with a dot) throughout the main text, figure captions, and figure labels to ensure consistency and adherence to the journal's formatting style. All relevant changes have been highlighted in the revised manuscript for your easy reference. We appreciate your careful attention to this detail, which has helped improve the overall presentation of our work.

**Comment 2:** Both terms "Arabidopsis thaliana" and "Arabidopsis" are used interchangeably. It is recommended to choose one term (e.g., "Arabidopsis") and use it consistently throughout the manuscript, clarifying at first mention that it refers to Arabidopsis thaliana.

**Response:** Thank you for this helpful suggestion. We have now standardized the usage throughout the manuscript. At the first mention in the Introduction, we have clearly stated "Arabidopsis thaliana" and thereafter used "Arabidopsis" consistently in all subsequent references. This approach follows standard scientific writing conventions and improves readability. We have revised all occurrences accordingly and highlighted the changes in the manuscript.

**Comment 3:** In the cis-element analysis figure, it is recommended to include a brief explanation in the legend indicating what each color represents, or ensure the colors are clearly labeled in the figure itself.

**Response:** Thank you for this constructive suggestion. We have now revised Fig 6 (the cis-element analysis figure) to include a comprehensive color legend directly in the figure caption. The detailed color legend is as follows: green, light-responsive; yellow, part of a light-responsive element; pink, MYB binding site (drought-inducibility); blue, palisade mesophyll cell differentiation; red, ABA-responsive; gray, meristem expression; light green, gapA-CMA1 light-responsive; orange, light-responsive regulatory; purple, module for light response. We believe this addition significantly improves the clarity and interpretability of the figure for readers.

**Comment 4:** Most references are well formatted, but a few inconsistencies remain, such as:

(1) Journal title abbreviations (e.g., Plant J vs Plant J.)

(2) Some references are missing volume/issue numbers or DOIs. It is recommended to double-check and complete these entries for consistency and completeness.

**Response:** Thank you for your careful review of our reference list. We have now thoroughly revised all references to ensure consistency and completeness:

**Reviewer #3:**

**Comment:** I have carefully reviewed the revised manuscript entitled "Genome-Wide Identification of tomato B-RRs Gene Family and Expression Analysis in Response to ABA and Drought" by Sun et al. This manuscript has undergone the first round of review. After a detailed reading of the revised version and the authors' responses to the comments from the two reviewers in the first round, I consider that the authors have effectively and comprehensively addressed all the comments and suggestions put forward by the previous reviewers. The quality of this revision was significantly improved. The authors have supplemented and improved the relevant experimental details, data interpretation and literature citation as required by the first-round reviewers, which makes the research conclusions more convincing. I have no new questions or additional suggestions for revision regarding the current version of the manuscript. The manuscript has met the publication requirements of PLOS ONE.

**Response:** We are sincerely grateful for your positive and encouraging evaluation of our revised manuscript. We greatly appreciate your time and effort in reviewing our work, and we are delighted to hear that you find our revisions satisfactory and that the manuscript now meets the publication requirements of PLOS ONE. Your affirmation is a great encouragement to us, and we thank you once again for your constructive feedback during the review process.

**Reviewer #4:**

**Comment:** (No Response)

**Response:** The authors appreciate the opportunity to submit this revised manuscript and acknowledge the valuable input from all reviewers. We regret that Reviewer #4 did not provide specific comments, but we trust that our comprehensive revisions have adequately addressed any potential concerns.

**Additional Note:**

We would like to express our sincere gratitude to the Academic Editor and all the reviewers for their insightful and constructive comments, which have significantly helped us improve the quality of our manuscript. We believe that all comments and suggestions have been fully addressed in this revised version. The manuscript has been thoroughly proofread and all changes have been clearly marked for easy identification.

We hope that the revised manuscript now meets the standards for publication in PLOS ONE and look forward to your favorable decision.
