# Supplementary material for: Genome-wide identification of tomato B-RRs gene family and expression analysis in response to ABA and drought
Source: PLoS One. 2026 Aug 17;21(8):e0356487. doi: 10.1371/journal.pone.0356487 (PMC13480614; doi:10.1371/journal.pone.0356487)
Supplement: Peer review history

# Peer review: Decision Letter 0

**DOI:** 10.1371/journal.pone.0356487.r001

**Authors:** Prafull Salvi

5 Dec 2025

PONE-D-25-29384

Genome-Wide Identification of tomato B-RRs Gene Family and Expression Analysis in Response to ABA and Drought

PLOS One

Dear Dr. Sun,

Thank you for submitting your manuscript to PLOS ONE. After careful consideration, we feel that it has merit but does not fully meet PLOS ONE’s publication criteria as it currently stands. Therefore, we invite you to submit a revised version of the manuscript that addresses the points raised during the review process.

Please submit your revised manuscript by Jan 19 2026 11:59PM. If you will need more time than this to complete your revisions, please reply to this message or contact the journal office at plosone@plos.org. When you're ready to submit your revision, log on to https://www.editorialmanager.com/pone/ and select the 'Submissions Needing Revision' folder to locate your manuscript file.

Please include the following items when submitting your revised manuscript:

- A rebuttal letter that responds to each point raised by the academic editor and reviewer(s). You should upload this letter as a separate file labeled 'Response to Reviewers'.
- A marked-up copy of your manuscript that highlights changes made to the original version. You should upload this as a separate file labeled 'Revised Manuscript with Track Changes'.
- An unmarked version of your revised paper without tracked changes. You should upload this as a separate file labeled 'Manuscript'.

If you would like to make changes to your financial disclosure, please include your updated statement in your cover letter. Guidelines for resubmitting your figure files are available below the reviewer comments at the end of this letter.

If applicable, we recommend that you deposit your laboratory protocols in protocols.io to enhance the reproducibility of your results. Protocols.io assigns your protocol its own identifier (DOI) so that it can be cited independently in the future. For instructions see: https://journals.plos.org/plosone/s/submission-guidelines#loc-laboratory-protocols. Additionally, PLOS ONE offers an option for publishing peer-reviewed Lab Protocol articles, which describe protocols hosted on protocols.io. Read more information on sharing protocols at https://plos.org/protocols?utm_medium=editorial-email&utm_source=authorletters&utm_campaign=protocols.

We look forward to receiving your revised manuscript.

Kind regards,

Prafull Salvi, PhD

Academic Editor

PLOS One

Journal Requirements:

When submitting your revision, we need you to address these additional requirements.

1.Please ensure that your manuscript meets PLOS ONE's style requirements, including those for file naming. The PLOS ONE style templates can be found at

https://journals.plos.org/plosone/s/file?id=wjVg/PLOSOne_formatting_sample_main_body.pdf and

https://journals.plos.org/plosone/s/file?id=ba62/PLOSOne_formatting_sample_title_authors_affiliations.pdf

2. PLOS requires an ORCID iD for the corresponding author in Editorial Manager on papers submitted after December 6th, 2016. Please ensure that you have an ORCID iD and that it is validated in Editorial Manager. To do this, go to ‘Update my Information’ (in the upper left-hand corner of the main menu), and click on the Fetch/Validate link next to the ORCID field. This will take you to the ORCID site and allow you to create a new iD or authenticate a pre-existing iD in Editorial Manager.

3. We note that the grant information you provided in the ‘Funding Information’ and ‘Financial Disclosure’ sections do not match.

When you resubmit, please ensure that you provide the correct grant numbers for the awards you received for your study in the ‘Funding Information’ section.

4. We note that your Data Availability Statement is currently as follows: “All relevant data are within the manuscript and its Supporting Information files.”

Please confirm at this time whether or not your submission contains all raw data required to replicate the results of your study. Authors must share the “minimal data set” for their submission. PLOS defines the minimal data set to consist of the data required to replicate all study findings reported in the article, as well as related metadata and methods (https://journals.plos.org/plosone/s/data-availability#loc-minimal-data-set-definition).

For example, authors should submit the following data:

- The values behind the means, standard deviations and other measures reported;

- The values used to build graphs;

- The points extracted from images for analysis.

Authors do not need to submit their entire data set if only a portion of the data was used in the reported study.

If your submission does not contain these data, please either upload them as Supporting Information files or deposit them to a stable, public repository and provide us with the relevant URLs, DOIs, or accession numbers. For a list of recommended repositories, please see https://journals.plos.org/plosone/s/recommended-repositories.

If there are ethical or legal restrictions on sharing a de-identified data set, please explain them in detail (e.g., data contain potentially sensitive information, data are owned by a third-party organization, etc.) and who has imposed them (e.g., an ethics committee). Please also provide contact information for a data access committee, ethics committee, or other institutional body to which data requests may be sent. If data are owned by a third party, please indicate how others may request data access.

5. We notice that your supplementary figures are uploaded with the file type 'Figure'. Please amend the file type to 'Supporting Information'. Please ensure that each Supporting Information file has a legend listed in the manuscript after the references list

6. If the reviewer comments include a recommendation to cite specific previously published works, please review and evaluate these publications to determine whether they are relevant and should be cited. There is no requirement to cite these works unless the editor has indicated otherwise.

Additional Editor Comments:

Dear Authors,

Your manuscript presents interesting findings, but it requires substantial methodological and analytical improvements before it can be considered further. I therefore invite you to submit a majorly revised version of the manuscript that fully addresses the issues raised. Please carefully consider and respond to all concerns outlined in the reviewers’ comments.

[Note: HTML markup is below. Please do not edit.]

Reviewer's Responses to Questions

**Comments to the Author**

1. Is the manuscript technically sound, and do the data support the conclusions?

The manuscript must describe a technically sound piece of scientific research with data that supports the conclusions. Experiments must have been conducted rigorously, with appropriate controls, replication, and sample sizes. The conclusions must be drawn appropriately based on the data presented.

Reviewer #1: Partly

Reviewer #2: Yes

**********

2. Has the statistical analysis been performed appropriately and rigorously?

Reviewer #1: Yes

Reviewer #2: Yes

**********

3. Have the authors made all data underlying the findings in their manuscript fully available?

The PLOS Data policy requires authors to make all data underlying the findings described in their manuscript fully available without restriction, with rare exception (please refer to the Data Availability Statement in the manuscript PDF file). The data should be provided as part of the manuscript or its supporting information, or deposited to a public repository. For example, in addition to summary statistics, the data points behind means, medians and variance measures should be available. If there are restrictions on publicly sharing data—e.g. participant privacy or use of data from a third party—those must be specified.

Reviewer #1: Yes

Reviewer #2: Yes

**********

4. Is the manuscript presented in an intelligible fashion and written in standard English?

PLOS ONE does not copyedit accepted manuscripts, so the language in submitted articles must be clear, correct, and unambiguous. Any typographical or grammatical errors should be corrected at revision, so please note any specific errors here.

Reviewer #1: No

Reviewer #2: Yes

**********

5. Review Comments to the Author

Please use the space provided to explain your answers to the questions above. You may also include additional comments for the author, including concerns about dual publication, research ethics, or publication ethics. (Please upload your review as an attachment if it exceeds 20,000 characters)

Reviewer #1: This study represents the genome-wide identification of the B-type Response Regulator (B-RR) gene family in tomato and analyzes its expression patterns in response to ABA and drought stress. However, the manuscript has several issues regarding methodological description, result interpretation, language expression, and figure quality, as detailed below:

1. In the Materials and Methods section, the authors mention "30 mol/L ABA", which is clearly an unreasonable concentration. Please verify and correct this to a plausible concentration (e.g., µM or nM).

2. The phylogenetic tree in the manuscript was constructed using only Arabidopsis and tomato. The limited species coverage may affect the comprehensiveness and reliability of evolutionary relationship inference. It is recommended to include more representative species to improve the analysis.

3. The Discussion section should be strengthened by better linking the findings to existing research. For instance, a more in-depth comparative analysis between the tissue-specific expression of tomato SlRR genes and the functions of their homologs in Arabidopsis and rice would help clarify their conservation and species-specificity.

4. The language is not fluent and contains numerous grammatical and wording errors. For example:

o Line 98: "Polesitilegicle (PEG)" should be "Polyethylene glycol (PEG)".

o "Tran-scription factors" should be "Transcription factors".

o It is recommended that the entire manuscript undergoes professional English editing or proofreading by a native speaker.

5. Figure 1 requires the addition of confidence scores (e.g., from the modeling software) to clarify the reliability of the predicted structures.

6. Figure 7 needs specific scale annotation for the TPM value range. The color gradient should be optimized to improve readability.

7. The explanation "lowercase letters indicate significance" in Figure 8 is insufficient. The specific statistical method used should be explicitly stated.

8. The image resolution is too low, resulting in blurred text.

9. In Table 2, what does "Nc a ; Nc b" represent? Please define these abbreviations in the table note to avoid ambiguity.

10. Lines 10-12, the statement "11, 7, 29, and 11 members" is vague. It is recommended to explicitly link the numbers to their respective species (Arabidopsis, rice, wheat, apple) and corresponding reference numbers, for example: "11, 7, 29, and 11 members have been identified in Arabidopsis [4], rice [5], wheat [6], and apple [7], respectively."

11. In the Keywords, "ABA response" and "Drought stress" are suggested to be adjusted to "ABA responsiveness" and "Drought tolerance" respectively, to align with common terminology in the field.

12. The formatting of reference citations is inconsistent; for instance, some references lack page numbers or volume/issue numbers.

Reviewer #2: B-type Response Regulators (B-RRs) play a crucial role in cytokinin signaling and stress response. Here authors identified a total of ten B-RR genes in the tomato (Solanum lycopersicum) genome, and analyzed their gene structure, chromosomal localization, phylogenic relationship, tissue-specific and drought-responsive expression pattern, and sequence motif. There are still some issues that need to be interrogated.

[1] The Abstract section might be confusing and should be reorganized. For example, its first sentence states a role for B-RRs gene family in cytokinin signal transduction, while its findings further demonstrate its involvement in ABA-mediated drought stress responses. Given the known antagonistic relationship between ABA and cytokinin, we are curious whether the authors have considered a potential role for this family in the crosstalk between these two hormonal pathways? It is essential to determine the cytokinin content in tomato plants under drought stress and ABA treatment conditions.

[2] The following aspects of the bioinformatically predicted results necessitate further validation through molecular experiments. Firstly, the subcellular localization predictions should be experimentally validated, for example, by transiently expressing GFP-fusion constructs in protoplasts or tobacco leaves. Secondly, the tissue-specific expression pattern identified by RNA-seq needs to be verified by RT-qPCR (Fig.7). Thirdly, the coding sequences (CDS) of the ten SlRR genes should be cloned and validated through Sanger sequencing. Additionally, the cloned SlRR CDSs should be deposited in public database, such as GenBase/NGDC (https://ngdc.cncb.ac.cn/genbase) and GenBank/NCBI (https://www.ncbi.nlm.nih.gov/genbank/).

[3] In the sentence ‘The protein sequences of the Arabidopsis B-ARRs family were obtained from the Tomato Genome Database (https://solgenomics.net/)’ (Line46-47), the source was incorrectly cited as ‘the Tomato Genome Database (https://solgenomics.net/)’ instead of the correct database, ‘the Arabidopsis Information Resource (TAIR, https://www.arabidopsis.org/)’.

[4] ‘an E-value threshold set as 1E-5’ was improperly written as ‘an E-value threshold set as -5’ (Line49).

[5] Is the multiple sequence alignment (MSA) performed using ClustalW or MUSCLE, as implemented in the MEGA7 packages? (The relevant text states, ‘The protein sequences of the Arabidopsis and the tomato B-RR family were aligned using MEGA7 software’, Line69-70).

[6] Please provide the website address for the NCBI website (https://www.ncbi.nlm.nih.gov/Structure/cdd/wrpsb.cgi, Line74), MEME online software (https://meme-suite.org/meme/, Line77), and PlantCARE online software (https://bioinformatics.psb.ugent.be/webtools/plantcare/html/, Line84).

[7] It is suggested that the B-RR genes from Oryza sativa be incorporated into the NJ-tree (Fig.4). Bootstrap sampling should be employed to construct the NJ-tree, and the bootstrapping values should be indicated on its branches.

[8] The common name, such as Thale cress or mouse-ear cress, should be used as an alternative to the non-italicized “Arabidopsis” (e.g., Line10, Line14, Line18, Line27, Line46, Line219, Line289, Line301, etc). This is necessary because Arabidopsis is a genus name and should be written in italics. Additionally, the species name Arabidopsis thaliana (Line246-247) should also be italicized to adhere to standard taxonomic converntions.

**********

6. PLOS authors have the option to publish the peer review history of their article (what does this mean?). If published, this will include your full peer review and any attached files.

If you choose “no”, your identity will remain anonymous but your review may still be made public.

**Do you want your identity to be public for this peer review?**  For information about this choice, including consent withdrawal, please see our Privacy Policy.

Reviewer #1: No

Reviewer #2: No

**********

[NOTE: If reviewer comments were submitted as an attachment file, they will be attached to this email and accessible via the submission site. Please log into your account, locate the manuscript record, and check for the action link "View Attachments". If this link does not appear, there are no attachment files.]

To ensure your figures meet our technical requirements, please review our figure guidelines: https://journals.plos.org/plosone/s/figures

You may also use PLOS’s free figure tool, NAAS, to help you prepare publication quality figures: https://journals.plos.org/plosone/s/figures#loc-tools-for-figure-preparation.

NAAS will assess whether your figures meet our technical requirements by comparing each figure against our figure specifications.

# Author comment: Author response to Decision Letter 1

**DOI:** 10.1371/journal.pone.0356487.r002

29 Jan 2026

Reviewer #1:

Comment 1: In the Materials and Methods section, the authors mention "30 mol/L ABA", which is clearly an unreasonable concentration. Please verify and correct this to a plausible concentration (e.g., µM or nM).

Response: Thank you for your careful review and for pointing out the error in the manuscript. The mention of “30 mol/L ABA” in the Materials and Methods section was an unintentional typo. We have corrected this to the appropriate experimental concentration, “30 µM ABA”, in the revised version of the manuscript. We appreciate your valuable comment, which has helped improve the accuracy of our work.

Comment 2: The phylogenetic tree in the manuscript was constructed using only Arabidopsis and tomato. The limited species coverage may affect the comprehensiveness and reliability of evolutionary relationship inference. It is recommended to include more representative species to improve the analysis.

Response: Thank you for your valuable suggestion. We agree that expanding the phylogenetic analysis to include more species would strengthen the evolutionary inference.

In response to your comment, we have now reconstructed the phylogenetic tree to include B-RR amino acid sequences from additional representative species, specifically rice (Oryza sativa) and apple (Malus domestica). This expanded analysis is included in the revised manuscript and provides a broader perspective on the evolutionary relationships within the B-RR gene family. We believe this revision has significantly improved the comprehensiveness and reliability of our phylogenetic analysis, and we appreciate your insightful feedback.

Comment 3: The Discussion section should be strengthened by better linking the findings to existing research. For instance, a more in-depth comparative analysis between the tissue-specific expression of tomato SlRR genes and the functions of their homologs in Arabidopsis and rice would help clarify their conservation and species-specificity.

Response: Thank you for the suggestion. We have revised the Discussion to include a detailed comparative analysis of tissue-specific expression among tomato, Arabidopsis, and rice homologs, clarifying their conservation and potential species-specific roles.

Comment 4: The language is not fluent and contains numerous grammatical and wording errors. For example:

o Line 98: "Polesitilegicle (PEG)" should be "Polyethylene glycol (PEG)".

o "Tran-scription factors" should be "Transcription factors".

o It is recommended that the entire manuscript undergoes professional English editing or proofreading by a native speaker.

Response: Thank you for identifying these language issues. We have corrected the specific errors noted and have now had the entire manuscript professionally edited to improve its fluency and grammar.

Comment 5: Figure 1 requires the addition of confidence scores (e.g., from the modeling software) to clarify the reliability of the predicted structures.

Response: Thank you for your suggestion. We have revised Figure 1 to include the requested confidence scores, with the detailed score distribution provided separately in the newly added Table 3.

Comment 6: Figure 7 needs specific scale annotation for the TPM value range. The color gradient should be optimized to improve readability.

Response: Thank you for your suggestion. We have now optimized Figure 7 by adding a specific scale annotation for the TPM value range and improving the color gradient to enhance overall readability.

Comment 7: The explanation "lowercase letters indicate significance" in Figure 8 is insufficient. The specific statistical method used should be explicitly stated.

Response: Thank you for your suggestion. We have updated Figure 8 to clearly state the specific statistical method used for the significance analysis.

Comment 8: The image resolution is too low, resulting in blurred text.

Response: Thank you for pointing out the issue with image clarity. We have regenerated and replaced all figures in the manuscript with high-resolution versions. The slight blur in the PDF may have been caused by file compression during formatting; we suggest downloading the original image files for a clearer view, as they are of high resolution.

Comment 9: In Table 2, what does "Nc a ; Nc b" represent? Please define these abbreviations in the table note to avoid ambiguity.

Response: Thank you for your feedback. We have now added a clear definition for "Nc a" and "Nc b" in the notes of Table 2 to resolve any potential ambiguity.

Comment 10: Lines 10-12, the statement "11, 7, 29, and 11 members" is vague. It is recommended to explicitly link the numbers to their respective species (Arabidopsis, rice, wheat, apple) and corresponding reference numbers, for example: "11, 7, 29, and 11 members have been identified in Arabidopsis [4], rice [5], wheat [6], and apple [7], respectively."

Response: Thank you for this suggestion. We have revised the sentence to clearly link each member count to its corresponding species and reference, as recommended.

Comment 11: In the Keywords, "ABA response" and "Drought stress" are suggested to be adjusted to "ABA responsiveness" and "Drought tolerance" respectively, to align with common terminology in the field.

Response: Thank you for the suggestions. We have updated the keywords as recommended, changing "ABA response" to "ABA responsiveness" and "Drought stress" to "Drought tolerance" to align with the field's standard terminology.

Comment 12: The formatting of reference citations is inconsistent; for instance, some references lack page numbers or volume/issue numbers.

Response: Thank you for your feedback. We have revised the reference list in accordance with the journal's formatting guidelines to ensure consistency, including the addition of missing volume, issue, and page numbers where applicable.

Reviewer #2:

Comment 1: The Abstract section might be confusing and should be reorganized. For example, its first sentence states a role for B-RRs gene family in cytokinin signal transduction, while its findings further demonstrate its involvement in ABA-mediated drought stress responses. Given the known antagonistic relationship between ABA and cytokinin, we are curious whether the authors have considered a potential role for this family in the crosstalk between these two hormonal pathways? It is essential to determine the cytokinin content in tomato plants under drought stress and ABA treatment conditions.

Response: Thank you for your insightful and constructive comment. In response, we have thoroughly revised the manuscript:The Abstract has been reorganized to present the rationale and findings more logically. A new section has been added to the Discussion. Here, we explicitly explore the potential role of the studied B-RRs in mediating the crosstalk between cytokinin and ABA signaling during drought stress, based on our expression pattern analysis and the established antagonistic relationship between the two hormones.Regarding your suggestion on cytokinin content measurement, we fully agree that it would be a valuable experiment. We plan to perform these measurements in our follow-up functional studies. In the revised discussion, we have incorporated this point as an important future research direction.

Comment 2: The following aspects of the bioinformatically predicted results necessitate further validation through molecular experiments. Firstly, the subcellular localization predictions should be experimentally validated, for example, by transiently expressing GFP-fusion constructs in protoplasts or tobacco leaves. Secondly, the tissue-specific expression pattern identified by RNA-seq needs to be verified by RT-qPCR (Fig.7). Thirdly, the coding sequences (CDS) of the ten SlRR genes should be cloned and validated through Sanger sequencing. Additionally, the cloned SlRR CDSs should be deposited in public database, such as GenBase/NGDC (https://ngdc.cncb.ac.cn/genbase) and GenBank/NCBI (https://www.ncbi.nlm.nih.gov/genbank/).

Response: Thank you for these constructive suggestions to strengthen the validation of our results.

In direct response to your recommendation, we have now performed RT-qPCR analysis, which successfully validates the tissue-specific expression patterns originally observed in the RNA-seq data (Fig. 7). These new results have been added to the revised manuscript.

We fully agree that experimental validation of subcellular localization and cloning of the CDS sequences, as you suggested, are important next steps. Given the current constraints of project timeline and resource availability—particularly nearing the conclusion of this study—we were unable to incorporate these additional experiments at this stage. However, we have explicitly acknowledged these points as key objectives for our future research in the revised Discussion section.We appreciate your guidance, which has significantly improved the present work and clearly outlines the path forward.

Comment 3: In the sentence ‘The protein sequences of the Arabidopsis B-ARRs family were obtained from the Tomato Genome Database (https://solgenomics.net/)’ (Line46-47), the source was incorrectly cited as ‘the Tomato Genome Database (https://solgenomics.net/)’ instead of the correct database, ‘the Arabidopsis Information Resource (TAIR, https://www.arabidopsis.org/)’.

Response: Thank you for identifying this oversight. The sentence has been corrected to accurately cite the source: "The protein sequences of the Arabidopsis B-ARRs family were obtained from The Arabidopsis Information Resource (TAIR, https://www.arabidopsis.org/)."

Comment 4: ‘an E-value threshold set as 1E-5’ was improperly written as ‘an E-value threshold set as -5’ (Line49).

Response: Thank you for pointing out this error. The text has been corrected to "an E-value threshold set as 1E-5" in the revised manuscript.

Comment 5: Is the multiple sequence alignment (MSA) performed using ClustalW or MUSCLE, as implemented in the MEGA7 packages? (The relevant text states, ‘The protein sequences of the Arabidopsis and the tomato B-RR family were aligned using MEGA7 software’, Line69-70).

Response: Thank you for raising this point. We have clarified the Materials and Methods section to specify that the multiple sequence alignment was performed using the ClustalW method within the MEGA7 software.

Comment 6: Please provide the website address for the NCBI website (https://www.ncbi.nlm.nih.gov/Structure/cdd/wrpsb.cgi, Line74), MEME online software (https://meme-suite.org/meme/, Line77), and PlantCARE online software (https://bioinformatics.psb.ugent.be/webtools/plantcare/html/, Line84).

Response: Thank you for your suggestion. We have updated the manuscript to include the full website addresses for the NCBI CD-search, MEME, and PlantCARE online tools.

Comment 7: It is suggested that the B-RR genes from Oryza sativa be incorporated into the NJ-tree (Fig.4). Bootstrap sampling should be employed to construct the NJ-tree, and the bootstrapping values should be indicated on its branches.

Response: Thank you for your valuable suggestion. We have now revised the phylogenetic tree (Fig. 4) by incorporating B-RR genes from Oryza sativa, and the bootstrap support values are indicated on the branches.

Comment 8: The common name, such as Thale cress or mouse-ear cress, should be used as an alternative to the non-italicized “Arabidopsis” (e.g., Line10, Line14, Line18, Line27, Line46, Line219, Line289, Line301, etc). This is necessary because Arabidopsis is a genus name and should be written in italics. Additionally, the species name Arabidopsis thaliana (Line246-247) should also be italicized to adhere to standard taxonomic converntions.

Response: Thank you for pointing this out. We have revised the manuscript throughout to ensure correct formatting.

Attachment Submitted filename: Response to reviewers.docx

# Peer review: Decision Letter 1

**DOI:** 10.1371/journal.pone.0356487.r003

**Authors:** Shailender Verma

26 May 2026

PONE-D-25-29384R1

Genome-Wide Identification of tomato B-RRs Gene Family and Expression Analysis in Response to ABA and Drought

PLOS One

Dear Dr. Sun,

Thank you for submitting your manuscript to PLOS ONE. After careful consideration, we feel that it has merit but does not fully meet PLOS ONE’s publication criteria as it currently stands. Therefore, we invite you to submit a revised version of the manuscript that addresses the points raised during the review process.

Please submit your revised manuscript by Jul 10 2026 11:59PM. If you will need more time than this to complete your revisions, please reply to this message or contact the journal office at plosone@plos.org. When you're ready to submit your revision, log on to https://www.editorialmanager.com/pone/ and select the 'Submissions Needing Revision' folder to locate your manuscript file.

Please include the following items when submitting your revised manuscript:

- A letter that responds to each point raised by the academic editor and reviewer(s). You should upload this letter as a separate file labeled 'Response to Reviewers'.
- A marked-up copy of your manuscript that highlights changes made to the original version. You should upload this as a separate file labeled 'Revised Manuscript with Track Changes'.
- An unmarked version of your revised paper without tracked changes. You should upload this as a separate file labeled 'Manuscript'.

If you would like to make changes to your financial disclosure, please include your updated statement in your cover letter. Guidelines for resubmitting your figure files are available below the reviewer comments at the end of this letter.

If applicable, we recommend that you deposit your laboratory protocols in protocols.io to enhance the reproducibility of your results. Protocols.io assigns your protocol its own identifier (DOI) so that it can be cited independently in the future. For instructions see: https://journals.plos.org/plosone/s/submission-guidelines#loc-laboratory-protocols. Additionally, PLOS ONE offers an option for publishing peer-reviewed Lab Protocol articles, which describe protocols hosted on protocols.io. Read more information on sharing protocols at https://plos.org/protocols?utm_medium=editorial-email&utm_source=authorletters&utm_campaign=protocols.

As the corresponding author, your ORCID iD is verified in the submission system and will appear in the published article. PLOS supports the use of ORCID, and we encourage all coauthors to register for an ORCID iD and use it as well. Please encourage your coauthors to verify their ORCID iD within the submission system before final acceptance, as unverified ORCID iDs will not appear in the published article. *Only*  the individual author can complete the verification step; PLOS staff *cannot*  verify ORCID iDs on behalf of authors.

We look forward to receiving your revised manuscript.

Kind regards,

Shailender Kumar Verma, Ph.D.

Academic Editor

PLOS One

Journal Requirements:

1. If the reviewer comments include a recommendation to cite specific previously published works, please review and evaluate these publications to determine whether they are relevant and should be cited. There is no requirement to cite these works unless the editor has indicated otherwise.

2. Please review your reference list to ensure that it is complete and correct. If you have cited papers that have been retracted, please include the rationale for doing so in the manuscript text, or remove these references and replace them with relevant current references. Any changes to the reference list should be mentioned in the rebuttal letter that accompanies your revised manuscript. If you need to cite a retracted article, indicate the article’s retracted status in the References list and also include a citation and full reference for the retraction notice.

[Note: HTML markup is below. Please do not edit.]

Reviewers' comments:

Reviewer's Responses to Questions

**Comments to the Author**

1. If the authors have adequately addressed your comments raised in a previous round of review and you feel that this manuscript is now acceptable for publication, you may indicate that here to bypass the “Comments to the Author” section, enter your conflict of interest statement in the “Confidential to Editor” section, and submit your "Accept" recommendation.

Reviewer #1: (No Response)

Reviewer #3: All comments have been addressed

Reviewer #4: All comments have been addressed

**********

2. Is the manuscript technically sound, and do the data support the conclusions?

The manuscript must describe a technically sound piece of scientific research with data that supports the conclusions. Experiments must have been conducted rigorously, with appropriate controls, replication, and sample sizes. The conclusions must be drawn appropriately based on the data presented.

Reviewer #1: Yes

Reviewer #3: Yes

Reviewer #4: Yes

**********

3. Has the statistical analysis been performed appropriately and rigorously?

Reviewer #1: Yes

Reviewer #3: Yes

Reviewer #4: Yes

**********

4. Have the authors made all data underlying the findings in their manuscript fully available?

The PLOS Data policy requires authors to make all data underlying the findings described in their manuscript fully available without restriction, with rare exception (please refer to the Data Availability Statement in the manuscript PDF file). The data should be provided as part of the manuscript or its supporting information, or deposited to a public repository. For example, in addition to summary statistics, the data points behind means, medians and variance measures should be available. If there are restrictions on publicly sharing data—e.g. participant privacy or use of data from a third party—those must be specified.

Reviewer #1: Yes

Reviewer #3: Yes

Reviewer #4: Yes

**********

5. Is the manuscript presented in an intelligible fashion and written in standard English?

PLOS ONE does not copyedit accepted manuscripts, so the language in submitted articles must be clear, correct, and unambiguous. Any typographical or grammatical errors should be corrected at revision, so please note any specific errors here.

Reviewer #1: Yes

Reviewer #3: Yes

Reviewer #4: Yes

**********

6. Review Comments to the Author

Please use the space provided to explain your answers to the questions above. You may also include additional comments for the author, including concerns about dual publication, research ethics, or publication ethics. (Please upload your review as an attachment if it exceeds 20,000 characters)

Reviewer #1: Although the manuscript has been carefully revised, a few sentences still read slightly redundantly or unnaturally. Minor adjustments are suggested:

1. The manuscript currently uses both Fig 1 and Fig. 1. It is recommended to choose one format and apply it consistently throughout (e.g., Fig 1 or Figure 1).

2. Both terms "Arabidopsis thaliana" and "Arabidopsis" are used interchangeably. It is recommended to choose one term (e.g., "Arabidopsis") and use it consistently throughout the manuscript, clarifying at first mention that it refers to Arabidopsis thaliana.

3. In the cis-element analysis figure, it is recommended to include a brief explanation in the legend indicating what each color represents, or ensure the colors are clearly labeled in the figure itself.

4. Most references are well formatted, but a few inconsistencies remain, such as:

(1) Journal title abbreviations (e.g., Plant J vs Plant J.)

(2) Some references are missing volume/issue numbers or DOIs. It is recommended to double-check and complete these entries for consistency and completeness.

Reviewer #3: I have carefully reviewed the revised manuscript entitled “Genome-Wide Identification of tomato B-RRs Gene Family and Expression Analysis in Response to ABA and Drought” by Sun et al. This manuscript has undergone the first round of review. After a detailed reading of the revised version and the authors’ responses to the comments from the two reviewers in the first round, I consider that the authors have effectively and comprehensively addressed all the comments and suggestions put forward by the previous reviewers. The quality of this revision was significantly improved.

The authors have supplemented and improved the relevant experimental details, data interpretation and literature citation as required by the first-round reviewers, which makes the research conclusions more convincing.

I have no new questions or additional suggestions for revision regarding the current version of the manuscript. The manuscript has met the publication requirements of PLOS ONE.

Reviewer #4: (No Response)

**********

7. PLOS authors have the option to publish the peer review history of their article (what does this mean?). If published, this will include your full peer review and any attached files.

If you choose “no”, your identity will remain anonymous but your review may still be made public.

**Do you want your identity to be public for this peer review?**  For information about this choice, including consent withdrawal, please see our Privacy Policy.

Reviewer #1: No

Reviewer #3: No

Reviewer #4: No

**********

[NOTE: If reviewer comments were submitted as an attachment file, they will be attached to this email and accessible via the submission site. Please log into your account, locate the manuscript record, and check for the action link "View Attachments". If this link does not appear, there are no attachment files.]

To ensure your figures meet our technical requirements, please review our figure guidelines: https://journals.plos.org/plosone/s/figures

You may also use PLOS’s free figure tool, NAAS, to help you prepare publication quality figures: https://journals.plos.org/plosone/s/figures#loc-tools-for-figure-preparation.

NAAS will assess whether your figures meet our technical requirements by comparing each figure against our figure specifications.

# Author comment: Author response to Decision Letter 2

**DOI:** 10.1371/journal.pone.0356487.r004

28 Jun 2026

Response to Reviewers

Manuscript Title: Genome-Wide Identification of tomato B-RRs Gene Family and Expression Analysis in Response to ABA and Drought

Manuscript ID: PONE-D-25-29384R1

Reviewer #1:

Comment 1: The manuscript currently uses both "Fig 1" and "Fig. 1". It is recommended to choose one format and apply it consistently throughout (e.g., "Fig 1" or "Figure 1").

Response: Thank you for pointing out this inconsistency. We have now carefully reviewed the entire manuscript and unified all figure citations to "Fig." (with a dot) throughout the main text, figure captions, and figure labels to ensure consistency and adherence to the journal's formatting style. All relevant changes have been highlighted in the revised manuscript for your easy reference. We appreciate your careful attention to this detail, which has helped improve the overall presentation of our work.

Comment 2: Both terms "Arabidopsis thaliana" and "Arabidopsis" are used interchangeably. It is recommended to choose one term (e.g., "Arabidopsis") and use it consistently throughout the manuscript, clarifying at first mention that it refers to Arabidopsis thaliana.

Response: Thank you for this helpful suggestion. We have now standardized the usage throughout the manuscript. At the first mention in the Introduction, we have clearly stated "Arabidopsis thaliana" and thereafter used "Arabidopsis" consistently in all subsequent references. This approach follows standard scientific writing conventions and improves readability. We have revised all occurrences accordingly and highlighted the changes in the manuscript.

Comment 3: In the cis-element analysis figure, it is recommended to include a brief explanation in the legend indicating what each color represents, or ensure the colors are clearly labeled in the figure itself.

Response: Thank you for this constructive suggestion. We have now revised Fig 6 (the cis-element analysis figure) to include a comprehensive color legend directly in the figure caption. The detailed color legend is as follows: green, light-responsive; yellow, part of a light-responsive element; pink, MYB binding site (drought-inducibility); blue, palisade mesophyll cell differentiation; red, ABA-responsive; gray, meristem expression; light green, gapA-CMA1 light-responsive; orange, light-responsive regulatory; purple, module for light response. We believe this addition significantly improves the clarity and interpretability of the figure for readers.

Comment 4: Most references are well formatted, but a few inconsistencies remain, such as:

(1) Journal title abbreviations (e.g., Plant J vs Plant J.)

(2) Some references are missing volume/issue numbers or DOIs. It is recommended to double-check and complete these entries for consistency and completeness.

Response: Thank you for your careful review of our reference list. We have now thoroughly revised all references to ensure consistency and completeness:

Reviewer #3:

Comment: I have carefully reviewed the revised manuscript entitled "Genome-Wide Identification of tomato B-RRs Gene Family and Expression Analysis in Response to ABA and Drought" by Sun et al. This manuscript has undergone the first round of review. After a detailed reading of the revised version and the authors' responses to the comments from the two reviewers in the first round, I consider that the authors have effectively and comprehensively addressed all the comments and suggestions put forward by the previous reviewers. The quality of this revision was significantly improved. The authors have supplemented and improved the relevant experimental details, data interpretation and literature citation as required by the first-round reviewers, which makes the research conclusions more convincing. I have no new questions or additional suggestions for revision regarding the current version of the manuscript. The manuscript has met the publication requirements of PLOS ONE.

Response: We are sincerely grateful for your positive and encouraging evaluation of our revised manuscript. We greatly appreciate your time and effort in reviewing our work, and we are delighted to hear that you find our revisions satisfactory and that the manuscript now meets the publication requirements of PLOS ONE. Your affirmation is a great encouragement to us, and we thank you once again for your constructive feedback during the review process.

Reviewer #4:

Comment: (No Response)

Response: The authors appreciate the opportunity to submit this revised manuscript and acknowledge the valuable input from all reviewers. We regret that Reviewer #4 did not provide specific comments, but we trust that our comprehensive revisions have adequately addressed any potential concerns.

Additional Note:

We would like to express our sincere gratitude to the Academic Editor and all the reviewers for their insightful and constructive comments, which have significantly helped us improve the quality of our manuscript. We believe that all comments and suggestions have been fully addressed in this revised version. The manuscript has been thoroughly proofread and all changes have been clearly marked for easy identification.

We hope that the revised manuscript now meets the standards for publication in PLOS ONE and look forward to your favorable decision.

Attachment Submitted filename: Response to Reviewers.doc

# Editor report: Decision Letter 2

**DOI:** 10.1371/journal.pone.0356487.r005

**Authors:** Shailender Kumar Verma

4 Aug 2026

Genome-Wide Identification of tomato B-RRs Gene Family and Expression Analysis in Response to ABA and Drought

PONE-D-25-29384R2

Dear Dr. Sun,

We’re pleased to inform you that your manuscript has been judged scientifically suitable for publication and will be formally accepted for publication once it meets all outstanding technical requirements.

Within one week, you’ll receive an e-mail detailing the required amendments. When these have been addressed, you’ll receive a formal acceptance letter and your manuscript will be scheduled for publication.

An invoice will be generated when your article is formally accepted. Please note, if your institution has a publishing partnership with PLOS and your article meets the relevant criteria, all or part of your publication costs will be covered. Please make sure your user information is up-to-date by logging into Editorial Manager at Editorial Manager® and clicking the ‘Update My Information' link at the top of the page. For questions related to billing, please contact billing support.

If your institution or institutions have a press office, please notify them about your upcoming paper to help maximize its impact. If they’ll be preparing press materials, please inform our press team as soon as possible -- no later than 48 hours after receiving the formal acceptance. Your manuscript will remain under strict press embargo until 2 pm Eastern Time on the date of publication. For more information, please contact onepress@plos.org.

Kind regards,

Shailender Kumar Verma, Ph.D.

Academic Editor

PLOS One

Additional Editor Comments (optional):

Reviewers' comments:

# Editor report: Acceptance letter

**DOI:** 10.1371/journal.pone.0356487.r006

**Authors:** Shailender Kumar Verma

PONE-D-25-29384R2

PLOS One

Dear Dr. Sun,

I'm pleased to inform you that your manuscript has been deemed suitable for publication in PLOS One. Congratulations! Your manuscript is now being handed over to our production team.

At this stage, our production department will prepare your paper for publication. This includes ensuring the following:

* All references, tables, and figures are properly cited

* All relevant supporting information is included in the manuscript submission,

* There are no issues that prevent the paper from being properly typeset

You will receive further instructions from the production team, including instructions on how to review your proof when it is ready. Please keep in mind that we are working through a large volume of accepted articles, so please give us a few days to review your paper and let you know the next and final steps.

Lastly, if your institution or institutions have a press office, please let them know about your upcoming paper now to help maximize its impact. If they'll be preparing press materials, please inform our press team within the next 48 hours. Your manuscript will remain under strict press embargo until 2 pm Eastern Time on the date of publication. For more information, please contact onepress@plos.org.

You will receive an invoice from PLOS for your publication fee after your manuscript has reached the completed accept phase. If you receive an email requesting payment before acceptance or for any other service, this may be a phishing scheme. Learn how to identify phishing emails and protect your accounts at https://explore.plos.org/phishing.

If we can help with anything else, please email us at customercare@plos.org.

Thank you for submitting your work to PLOS ONE and supporting open access.

Kind regards,

PLOS ONE Editorial Office Staff

on behalf of

Dr. Shailender Kumar Verma

Academic Editor

PLOS One
